# Phase II Study of Irinotecan, Trifluridine/tipiracil (TAS-102) plus Bevacizumab as a Later-line Therapy for Patients with Metastatic Colorectal Cancer (mCRC): a prospective single-center explorative study

**DOI:** 10.1038/s41416-024-02885-3

**Published:** 2024-10-24

**Authors:** Baoqi Li, Wenwei Yang, Na Liu, Deying Bi, Tingting Yang, Guifu Wu, Yongkun Sun

**Affiliations:** 1 Department of Medical Oncology, Beijing Chaoyang District Sanhuan Cancer Hospital, Beijing, 100122 China; 2 Department of Medical Oncology, National Cancer Center/National Clinical Research Center for Cancer/Cancer Hospital, Chinese Academy of Medical Sciences and Peking Union Medical College, Beijing, 100021 China

**Keywords:** Colon cancer, Phase II trials

## Abstract

**Purpose:**

To explore the efficacy and safety of the combination of irinotecan, trifluridine/tipiracil (TAS-102), and bevacizumab in a later‐line setting for metastatic colorectal cancer (mCRC) patients.

**Patients and methods:**

This was a single-center, phase II trial. The mCRC patients who are refractory to standard first-line and second-line treatment are eligible. Patients who previously received irinotecan while progressing during maintenance therapy are also eligible. The primary endpoint was the objective response rate (ORR).

**Results:**

Between August 1, 2022, and September 30, 2023, 35 patients were enrolled, and 31 of them were evaluable for efficacy. The ORR was 25.8% (8/31), and the disease control rate (DCR) was 93.5% (29/31). As of April 30, 2024, the median progression-free survival (PFS) was 9.2 months (95% CI 6.285-12.115), whereas the median overall survival (OS) was not reached with the 1-year OS rate of 73.5%. The most common grade 3/4 treatment-related adverse events were neutropenia (34.3%), anemia (17.1%), and thrombocytopenia (8.6%).

**Conclusion:**

Irinotecan, TAS-102 plus bevacizumab regimen preliminarily demonstrated promising efficacy with tolerable toxicity for mCRC patients as later‐line treatment. This regimen warrants further exploration in refractory mCRC patients.

## Introduction

Colorectal cancer (CRC) ranks as the second leading cause of cancer-related mortality and the third most common malignancy globally [1]. Due to the insidious onset of colorectal cancer, the majority of patients are in the late stage when it is diagnosed and lose the opportunity for radical surgery. Approximately half of the patients who experienced radical resection might subsequently develop recurrence. Patients with metastatic colorectal cancer (mCRC) patients have a poor prognosis with a five-year survival rate of about 12% [2]. For 95% of mCRC cases, which are microsatellite stable (MSS), chemotherapy is considered the mainstay in the treatment [3]. Chemotherapy is based on fluoropyrimidines (e.g., 5-fluorouracil and capecitabine) combined with oxaliplatin or irinotecan or both, which is the standard first- or second-line regimen for mCRC [4]. Furthermore, the addition of molecularly targeted agents, such as the vascular endothelial growth factor inhibitor (bevacizumab) and epidermal growth factor inhibitor (e.g., cetuximab or panitumumab), to the chemotherapy regimen, has been confirmed to significantly prolong the median survival time of patients with mCRC [5, 6]. Currently, several phase II studies indicated that anti-human epidermal growth factor receptor 2 (HER2) therapy could bring survival benefits for mCRC patients. Moreover, immunotherapy has revolutionized the treatment of mCRC with high microsatellite instability, bringing patients long-lasting responses [7].

In the third-line or beyond setting, several novel agents have been approved for refractory mCRC these years, such as regorafenib, TAS-102, and fruquintinib [8–10]. However, few available third- or later-line therapies with limited survival benefit make it hard to meet clinical needs. Therefore, designing an optimal third- or later-line treatment regimen to improve the response rate and prolong the survival of patients with mCRC is urgently needed. In third- or later-line settings, researchers are constantly exploring more intense regimens for mCRC patients. The antiangiogenic tyrosine kinase inhibitors (TKI) plus PD-1/PD-L1 blockade regimen have been beginning to receive attention since the REGONIVO study [11], while other studies involving TKI combined with PD-1/PD-L1 blockade did not bring promising results. In recent years, precision medicine and the intestinal bacteria flora have become a hotspot in CRC treatment.

TAS-102 is a novel, oral antitumor agent with a fixed-dose combination of trifluridine (FTD; α, α, α-trifluorothymidine) and tipiracil hydrochloride (TPI). FTD is a fluorinated thymidine analog with its efficacy against 5-FU-resistant tumors [12]. FTD could be directly incorporated into DNA strands and cause DNA dysfunction, contributing to antitumor effects [13]. However, FTD undergoes rapid degradation by thymidine phosphorylase, leading to a short half-life, which is the main obstacle to its clinical application. TPI, a potent inhibitor of thymidine phosphorylase, could markedly improve the bioavailability of FTD after administration [14]. With its unique mechanism of action, TAS-102 has proven its antitumor activity against 5-FU-resistant tumors in several preclinical research, which provided the rationale for its clinical applications. Based on several large-scale clinical trials (e.g., RECOURSE study, TERRA study), TAS-102 monotherapy could bring survival benefits to mCRC patients and was approved for mCRC third-line or beyond treatment [9, 15].

Nevertheless, the efficacy of TAS-102 monotherapy was limited compared to its combination regimens. Previous preclinical research preliminarily demonstrated that TAS-102 exhibited synergistic effects when combined with irinotecan or bevacizumab in colon cancer cells and human colorectal cancer xenograft models [16–18]. In recent years, TAS-102 plus bevacizumab regimen showed better efficacy in several phase II clinical trials, such as BiTS and TAS-CC3 study [19–22]. In 2023, the results of the phase III SUNLIGHT study demonstrated that TAS-102 plus bevacizumab could bring longer overall survival with tolerable toxicity for patients with refractory mCRC than TAS-102 alone [23]. Therefore, TAS-102 plus bevacizumab regimen has been approved for mCRC third-line treatment. The conventional frequency of TAS-102 administration is on days 1-5 and 8-12, repeated every four weeks. However, severe hematological toxicity hindered its application. In 2020, BiTS study indicated that biweekly TAS-102 (twice daily on days 1-5, every two weeks) with bevacizumab could bring similar antitumor activity but with better safety [22]. Irinotecan, a topoisomerase I inhibitor, is a classic chemotherapy drug that is the standard therapy for mCRC. For mCRC patients who were not exposed to irinotecan or previously received irinotecan while progressing during maintenance therapy, irinotecan might be a feasible drug.

Based on the studies mentioned above, the combination regimen of TAS-102, irinotecan, and bevacizumab might become a feasible third-line or later treatment option for mCRC. This phase II study was aimed to evaluate the efficacy and safety of the combination of TAS-102, irinotecan, and bevacizumab for patients with mCRC refractory to prior first- and second-line standard treatment.

## Patients and methods

### Study design

This single-arm, open-label, phase II study was conducted at Cancer Hospital Chinese Academy of Medical Sciences and Peking Union Medical College. This study aims to explore the efficacy and safety of the combination of irinotecan, TAS-102, and bevacizumab in a third‐line or later therapy for patients with mCRC. The mCRC patients who are refractory to first-line or second-line therapies without exposure to irinotecan are eligible. Patients who previously received irinotecan while progressing during maintenance therapy are also eligible. These patients received an intravenous infusion of irinotecan (150 mg/m^2^ on day 1) plus bevacizumab (5 mg/kg on day 1) and an oral administration of TAS-102 (30 mg/m^2^ given bid on days 1-5), repeated every 14 days. Treatment was continued until RECIST-defined or clinical disease progression, unacceptable toxicity, or patient withdrawal. Dose reductions or delays were allowed for significant treatment-associated toxicities (≥ grade 3 non-hematological or grade 4 hematological toxicity). The efficacy was assessed every three cycles. The primary endpoint was the objective response rate (ORR); secondary endpoints included progression-free survival (PFS), overall survival (OS), and safety. ORR was defined as the percentage of patients experiencing complete response (CR) and partial response (PR) according to RECIST version 1.1. PFS was defined as the time from the initial administration of this study medication to the date of the first documented disease progression or death. OS was defined as the time from the date of beginning receiving this study medication to the date of death.

The study adhered to the Declaration of Helsinki and Good Clinical Practice guidelines. Written informed consent was obtained from all participants. The Ethics Committee of the participating institution, the independent ethics committee of Beijing Chaoyang Sanhuan Cancer Hospital, approved this study. This study was registered on the Clinical Trials website (NCT06403709).

### Patient eligibility

The study’s key inclusion criteria were: (1) pathologically confirmed adenocarcinoma of the colon or rectum; (2) clinically diagnosed with metastatic CRC based on computed tomography (CT) and magnetic resonance imaging (MRI) (based on AJCC 8th edition); (3) the presence of at least one measurable lesion according to RECIST version 1.1. (4) Eastern Cooperative Oncology Group Performance Status (ECOG PS) of 0-2; (5) disease progression or intolerance after first-line and second-line treatment; (6) no prior exposure to irinotecan or previously received irinotecan while progressed during maintenance therapy; (7) age of 18-75 years old; (8) sufficient organ function. The main exclusion criteria were as follows: (1) hypersensitive to irinotecan, TAS-102, or bevacizumab; (2) previous tumor progression during irinotecan treatment, while patients progressed during maintenance therapy are eligible; (3) patients with active gastrointestinal bleeding; (4) prior surgery or other antitumor treatment conducted within four weeks before enrollment (except biopsy); (5) patients with a second primary malignant tumor; (6) severe concurrent diseases or medical conditions; (7) were pregnant or lactating; (8) deemed unsuitable as research subjects by the investigator.

### Efficacy and safety assessment

Efficacy assessment was performed at baseline (within 14 days before initiation of treatment) and every three cycles (6 weeks) or as clinically necessary by imaging modalities, including magnetic resonance imaging (MRI), computed tomography (CT), and/or positron-emission tomography (PET-CT). The investigator assessed the response using the Response Evaluation Criteria in Solid Tumors (RECIST) version 1.1. Patients who received at least one treatment cycle and had undergone radiologic/clinical progression assessments were evaluated for efficacy assessment. Safety assessments were conducted in all patients who were administered at least one dose of the study medication from the initial administration until 30 days after the final dose of the study drug. Adverse events (AEs) were graded based on the National Cancer Institute (NCI) Common Terminology Criteria for Adverse Events (CTCAE), version 5.0.

### Statistical analyses

The study was designed as an exploratory pilot study with an enrollment target of 35 patients. Descriptive statistics were used to summarize patient baseline characteristics, efficacy, and safety data. The Kaplan-Meier method was used to analyze the median follow-up period and survival endpoints (i.e., PFS and OS), expressed as medians with 95% CIs indicated, and establish the survival curves. The statistical analyses were performed using software SPSS version 29.0 (IBM Corp., Armonk, NY, United States) and GraphPad Prism 10 (GraphPad Software, Inc.). A two-sided *p*-value < 0.05 was considered statistically significant.

## Results

### Patient characteristics

A total of 35 patients with mCRC were enrolled from August 2022 to September 2023. All participants received at least one dose of the study drug. The median age was 58 years (from 35 to 79), and 51.4% of the patients were male. The majority (82.9%) of the patients had an ECOG PS score of 0-1. Regarding the degree of differentiation, 54.3% of the cases were medium differentiation, followed by medium-low differentiation (5.7%) and high-moderate differentiation (2.9%). Left-sided colon cancer was dominant (80.0%) in this group of patients. As for metastatic sites, 24 patients (68.6%) had liver metastasis, 21 (60%) had retroperitoneal or celiac lymph node metastasis, and 16 (45.7%) had lung metastasis. Twenty-one patients (60%) had two or more metastatic sites. Baseline RAS/BRAF gene status analysis was performed on 31 participants, and RAS/BRAF mutation occurs in 48.6% of enrolled patients. Fifteen patients harbored KRAS mutations, one had NRAS mutations, and one had BRAF gene mutation. Eighty percent of patients examined microsatellite status, and only one was dMMR/MSI-H. Regarding the number of lines of systemic therapy, among these 35 patients, 14 (40%) received this study regimen as third-line therapy and 21 (60%) as fourth-line or later therapy. Notably, most patients (28 cases, 80%) received this study regimen at the time over 18 months from diagnosis of first metastases. Moreover, 94.3% of patients had previously received irinotecan. In terms of prior targeted therapy, 33 patients (94.3%) had a history of bevacizumab administration, and 9 (25.7%) received anti-epidermal growth factor receptor monoclonal antibodies in previous treatment. Table 1 describes the baseline patients’ characteristics.

**Table 1:** Patient characteristics at baseline

Characteristic	No. of patients	%
**Entire population**	35	100.0
**Median (range) age, years**	58 (35–79)	
**Sex**		
Male	18	51.4
Female	17	48.6
**ECOG Performance Status**		
0	2	5.7
1	27	77.1
2	6	17.2
**Degree of differentiation**		
High-moderate differentiation	1	2.9
Medium differentiation	19	54.3
Medium-low differentiation	2	5.7
NA	13	37.1
**Primary tumor site**		
Left-sided	28	80.0
Right-sided	7	20.0
**Metastases**		
Retroperitoneal or celiac lymph node metastasis	21	60.0
Liver metastasis	24	68.6
Lung metastasis	16	45.7
≥ 2 metastatic sites	21	60.0
**Prior targeted therapy**		
Bevacizumab	33	94.3
Cetuximab	9	25.7
**Number of lines of treatment**		
Third-line	14	40.0
Fourth-line or later	21	60.0
**Time from diagnosis of first metastasis to randomization**		
≥ 18 months	28	80.0
< 18 months	7	20.0
**Prior exposure to irinotecan**		
Yes	33	94.3
No	2	5.7
**Microsatellite status**		
pMMR/MSS	27	77.1
dMMR/MSI-H	1	2.9
NA	7	20.0
**RAS/BRAF status**		
Wild type	14	40.0
Mutant type	17	48.6
NA	4	11.4

*ECOG* Eastern Cooperative Oncology Group, *NA* not available

### Efficacy

**Fig. 1:**
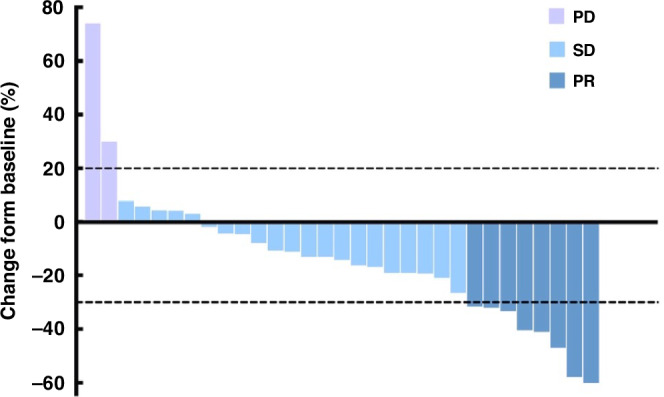
Clinical Efficacy. Waterfall plot of maximum percentage change in tumor size.

**Fig. 2:**
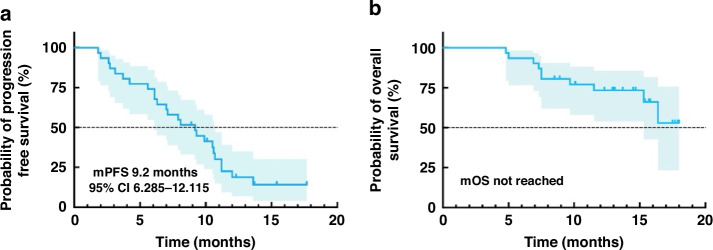
Survival Outcomes. Kaplan-Meier curves for progression-free survival (**a**) and overall survival (**b**).

As of April 30, 2024, 31 of 35 patients were evaluable for efficacy (1 case did not have a target lesion after radiofrequency ablation; 2 cases gave up treatment due to COVID-19; 1 was lost to follow-up). The median number of cycles administered was 9 (ranging from 1 to 19). Regarding short-term efficacy, as shown in Table 2, 8 patients achieved PR, 21 experienced SD, and two obtained progression disease (PD). The ORR was 25.8% (8/31), and the disease control rate (DCR) was 93.5% (29/31). Among the 21 patients with SD, 16 patients experienced tumor shrinkage. A waterfall plot of the maximum tumor shrinkage from baseline in 31 patients is shown in Fig. 1. As of April 2024, the median follow-up period was 14.5 months (95% confidence interval [CI], 13.337–15.663). The six-month PFS was 74.2% (23/31). The median PFS was 9.2 months (95%CI 6.285–12.115) (Fig. 2), whereas the median OS was not reached. The 1-year OS rate was 73.5%. At the time of analysis, ten patients have died, and 21 patients are still alive.

**Table 2:** Efficacy outcome

Variable	No. of patients (*n* = 31)	%
**CR**	0	0
**PR**	8	25.8
**SD**	21	67.7
**PD**	2	6.5
**ORR,** ***n*** **(%)**	8	25.8
**DCR,** ***n*** **(%)**	29	93.5
**Six-month PFS rate, (%)**		74.2
**One-year OS rate, (%)**		73.5
**PFS, median (95%CI), months**	9.2 (6.285–12.115)	
**OS, median (95%CI), months**	Not reached	

*CR* Complete response, *PR* partial response, *SD* stable disease, *PD* progressive disease, *PFS* progression-free survival, *OS* overall survival, *CI* confidence interval, *NR* not reached

### Safety

All of the 35 patients were evaluable for safety assessment. Each patient experienced one or more treatment-related adverse events (TRAEs) of any grade. Grade 3 or 4 TRAEs occurred in 17 patients (48.6%). The most common TRAEs were bone marrow suppression and gastrointestinal-related, such as nausea (94.3%), neutropenia (77.1%), anemia (51.4%), and thrombocytopenia (42.9%). Nausea and vomiting events were all grade 2 or lower. The most frequently occurring grade 3/4 TRAEs were hematologic toxicity: neutropenia (34.3%), anemia (17.1%), and thrombocytopenia (8.6%). Four patients developed grade 3/4 neutropenia and anemia, and two developed grade 3/4 neutropenia and thrombocytopenia. For five patients, the dosage of the drug was reduced because of severe adverse effects. Two patients experienced a dose increase due to improved physical performance after treatment. With supportive treatment, dose reduction, and/or temporary interruption, most TRAEs were reversible and manageable. No patients discontinued the study due to treatment-related adverse events. None of the patients died because of treatment-related AEs. Commonly encountered TRAEs are listed in Table 3.

**Table 3:** Most common adverse events in 35 participants (maximum grade per patient per event)

Adverse event	Any grade, *n* (%)	Grade 3 or 4, *n* (%)
**Hematologic toxicity**		
Anemia	18 (51.4)	6 (17.1)
Thrombocytopenia	15 (42.9)	3 (8.6)
Neutropenia	27 (77.1)	12 (34.3)
Febrile neutropenia	0	0
**Non-hematologic toxicity**		
Nausea	33 (94.3)	0
Vomiting	10 (28.6)	0
Diarrhea	6 (17.1)	1 (2.9)
Constipation	0	0
Hypertension	4 (11.4)	0
Fatigue	29 (82.9)	1 (2.9)
Hand-foot syndrome	0	0
Proteinuria	4 (11.4)	0
ALT/AST increased	8 (22.9)	1 (2.9)

## Discussion

In recent years, despite the research progress on colorectal cancer, there are limited therapeutic options for refractory mCRC. Therefore, more novel treatment regimens are warranted. This prospective pilot phase II trial preliminarily demonstrated the efficacy and safety of TAS-102 in combination with irinotecan and bevacizumab among patients with mCRC in third-line or later settings. Regarding the primary endpoint, this combination achieved an ORR of 25.8% with a median PFS of 9.2 months, exceeding the approved regimens. To our knowledge, this is the first clinical study to evaluate the efficacy and tolerability of such a combination in refractory mCRC patients for third- or later-line treatment.

For refractory mCRC, regorafenib, fruquintinib, and TAS-102 had been approved for third-line or later treatment. All these drugs demonstrated a superior effect over placebo, while their efficacy was still limited and difficult to meet clinical needs. The median OS ranged from 6.4 to 9.3 months, with a hazard ratio of 0.55–0.79 [8–10, 15, 24, 25]. Subsequently, TAS-102 was evaluated as a combination therapy with bevacizumab in a series of studies. Several studies of TAS-102 and bevacizumab regimen, including six prospective studies [19–23, 26] and three retrospective studies [27–29], showed that PFS ranged from 3.7 to 6.8 months and OS ranged from 8.6 to 14.4 months. ORR and DCR of these studies were 0.0-6.3% and 53.3–76.1%, respectively. However, among these studies, the OS only in two retrospective studies was longer than 14 months. OS in all prospective studies was less than 12 months. These studies also confirmed that the efficacy of biweekly TAS-102 and bevacizumab regimen was equivalent to that of conventional TAS-102 and bevacizumab therapy.

In the present study, the median PFS was 9.2 months, and the median OS was not reached. ORR and DCR were 25.8% and 93.5%, respectively. These efficacy data are superior to previous studies examining mCRC patients treated with approved third- or later-line therapies. Several reasons might account for the promising efficacy. Firstly, of this group of patients, 94.3% previously received irinotecan. These patients progressed during maintenance therapy, which indicated they were more sensitive to irinotecan treatment. Therefore, the improved efficacy of this combination might be attributed to patients’ high sensitivity to irinotecan. Moreover, the time from the diagnosis of the first metastasis until enrollment was 18 months or longer in 80% of this group of patients, which is higher than that reported in the SUNLIGHT study (57.5%) [23]. This baseline characteristic indicated that these patients had tumors with relatively indolent biological behavior and a slow progression rate. Thus, they might have a better prognosis.

Concerning safety, all-grade TRAEs occurred in all patients, and grade 3/4 TRAEs occurred in 48.6% of them. The most common TRAEs were bone marrow suppression and gastrointestinal-related. Neutropenia, anemia, and thrombocytopenia were the most frequently happening grade 3/4 TRAEs. The toxicity of this combination was manageable and well-tolerated, and even two patients experienced a dose increase during the treatment period because of their improved physical performance status. Most adverse events were reversible and within our expectations, and no TRAE led to treatment discontinuation or treatment-related deaths. In previous studies, the safety profile of TAS-102 monotherapy, TAS-102 plus bevacizumab regimen, or other irinotecan containing doublet or triplet combination [19, 21–23, 26, 30–36] showed the incidence of any-grade neutropenia ranged from 46% to 100%, and grade 3/4 neutropenia was 15.9%–100%. The reported incidence of all-grade anemia ranged from 28.9% to 89%, and the incidence in grades ≥ 3 was 4%–16%. The rate of any-grade thrombocytopenia was 14%–70%, and grade 3/4 thrombocytopenia was 0–12.5%. The incidence of hematologic toxicity in our study is consistent with those reported in previous studies. Notably, previous studies of irinotecan-containing regimens reported that the incidence of all-grade diarrhea was 38%–99.2% and the incidence in grades ≥ 3 was 0–83.4%, while our study showed decreased diarrhea events compared with those studies. The good tolerability of this triplet combination was also confirmed by a phase I dose-escalation study conducted by our team in the second-line setting (not published).

For mCRC patients who experienced multi-line treatments, cumulative drug toxicity might be established, and patients’ tolerance and toxicity profiles should be considered in treatment selection. In our study, more than half of patients received this medical regimen as a fourth-line or later treatment. Moreover, the number of metastatic sites also impacted survival. Patients who have multiple metastatic sites were demonstrated to experience shorter survival than patients with oligometastatic disease [37]. In our study, 60% of patients had ≥ 2 metastatic sites and tended to have a poor prognosis. Furthermore, 17.1% of patients had ECOG PS of 2. Patients with poor performance status (ECOG PS ≥ 2) are unlikely to tolerate the side effects of chemotherapy. Although the baseline characteristics of this group of patients indicated that they might have poor tolerance to systematic treatment and poor prognoses, the results showed encouraging efficacy and good tolerability. The results of this study have several implications for clinical practice. For refractory mCRC patients without a validated therapeutic target, there is an urgent need to find an effective regimen. Even in later-line settings, patients sensitive to previous treatment need a high intensity treatment. According to a randomized phase II study, the CAPability-01 trial, combining the PD-1monoclonal antibody sintilimab and the histone deacetylase inhibitor (HDACi) chidamide with anti-vascular endothelial growth factor (VEGF) monoclonal antibody bevacizumab might be a new direction for further exploration [38]. The triplet combination exhibited significantly improved outcomes compared to the doublet arm (sintilimab plus chidamide), with a greater PFS rate at 18 weeks (64.0% vs. 21.7%, *P* = 0.003), higher ORR (44.0% vs. 13.0%, *P* = 0.027) and longer median PFS (7.3 months vs. 1.5 months, *P* = 0.006). Based on our study, combining TAS-102, irinotecan, and bevacizumab might be another promising intensified regimen for refractory mCRC patients.

The present study has certain limitations. First, it is a single-arm study, lacking a randomized controlled cohort to directly compare the efficacy of our regimen with traditional third-line therapy. Second, the number of cases included in this study was relatively small, which might lead to selection bias. The patient selection was also impacted by patients’ prior irinotecan toxicity. Third, the short follow-up period may have swayed the reliability of the results to some extent. Fourth, this study was conducted exclusively among Chinese patients. Moreover, several patients were treated irregularly due to the impact of the COVID-19 epidemic. Therefore, the results of this study should be interpreted with caution.

As for future research directions, we plan to expand the cohort, extend the follow-up period, and systematically collect and analyze follow-up data to carry out a comprehensive evaluation of the long-term efficacy and safety of the novel regimen. Furthermore, we intend to design a large-scale multi-center phase II trial to further evaluate the efficacy and safety of this regimen. We also investigated the efficacy and safety of this regimen in a second-line setting, and the results are eagerly awaited.

In conclusion, this phase II study revealed that the combination regimen of TAS-102, irinotecan, and bevacizumab showed promising efficacy and manageable safety profiles in mCRC patients who refractory to standard treatment. Although further studies are needed, this novel regimen might be a promising alternative option for colorectal cancer patients who will receive therapy in a later-line setting. It is necessary to conduct further research to obtain more reliable data, providing a practical reference for clinical treatment.

## Data Availability

The data that support the findings of this study are available from the corresponding author upon reasonable request.

## References

[1] Sung H, Ferlay J, Siegel RL, Laversanne M, Soerjomataram I, Jemal A, et al. Global Cancer Statistics 2020: GLOBOCAN Estimates of Incidence and Mortality Worldwide for 36 Cancers in 185 Countries. CA Cancer J Clin. 2021;71:209–49. 33538338 10.3322/caac.21660

[2] Miller KD, Nogueira L, Mariotto AB, Rowland JH, Yabroff KR, Alfano CM, et al. Cancer treatment and survivorship statistics, 2019. CA Cancer J Clin. 2019;69:363–85. 31184787 10.3322/caac.21565

[3] Lin S, Chen W, Chen Z, Liang J, Zhong L, Jiang M. Efficacy of sintilimab and fruquintinib combination treatment in the management of microsatellite-stable metastatic colorectal cancer: a case report. Ann Transl Med. 2022;10:380. 35433988 10.21037/atm-22-359 PMC9011267

[4] Ohishi T, Kaneko MK, Yoshida Y, Takashima A, Kato Y, Kawada M. Current Targeted Therapy for Metastatic Colorectal Cancer. Int J Mol Sci. 2023;24:1702. 10.3390/ijms24021702 PMC9864602 36675216

[5] Venook AP, Niedzwiecki D, Lenz HJ, Innocenti F, Fruth B, Meyerhardt JA, et al. Effect of First-Line Chemotherapy Combined With Cetuximab or Bevacizumab on Overall Survival in Patients With KRAS Wild-Type Advanced or Metastatic Colorectal Cancer: A Randomized Clinical Trial. Jama. 2017;317:2392–401. 28632865 10.1001/jama.2017.7105 PMC5545896

[6] Gao M, Jiang T, Li P, Zhang J, Xu K, Ren T. Efficacy and safety of HER2-targeted inhibitors for metastatic colorectal cancer with HER2-amplified: A meta-analysis. Pharm Res. 2022;182:106330. 10.1016/j.phrs.2022.106330 35781058

[7] Hou W, Yi C, Zhu H. Predictive biomarkers of colon cancer immunotherapy: Present and future. Front Immunol. 2022;13:1032314. 36483562 10.3389/fimmu.2022.1032314 PMC9722772

[8] Grothey A, Van Cutsem E, Sobrero A, Siena S, Falcone A, Ychou M, et al. Regorafenib monotherapy for previously treated metastatic colorectal cancer (CORRECT): an international, multicentre, randomised, placebo-controlled, phase 3 trial. Lancet. 2013;381:303–12. 23177514 10.1016/S0140-6736(12)61900-X

[9] Mayer RJ, Van Cutsem E, Falcone A, Yoshino T, Garcia-Carbonero R, Mizunuma N, et al. Randomized trial of TAS-102 for refractory metastatic colorectal cancer. N. Engl J Med. 2015;372:1909–19. 25970050 10.1056/NEJMoa1414325

[10] Li J, Qin S, Xu RH, Shen L, Xu J, Bai Y, et al. Effect of Fruquintinib vs Placebo on Overall Survival in Patients With Previously Treated Metastatic Colorectal Cancer: The FRESCO Randomized Clinical Trial. Jama. 2018;319:2486–96. 29946728 10.1001/jama.2018.7855 PMC6583690

[11] Fukuoka S, Hara H, Takahashi N, Kojima T, Kawazoe A, Asayama M, et al. Regorafenib Plus Nivolumab in Patients With Advanced Gastric or Colorectal Cancer: An Open-Label, Dose-Escalation, and Dose-Expansion Phase Ib Trial (REGONIVO, EPOC1603). J Clin Oncol. 2020;38:2053–61. 32343640 10.1200/JCO.19.03296

[12] Chakrabarti S, Wintheiser G, Tella SH, Oxencis C, Mahipal A. TAS-102: A resurrected novel Fluoropyrimidine with expanding role in the treatment of gastrointestinal malignancies. Pharm Ther. 2021;224:107823. 10.1016/j.pharmthera.2021.107823 33667525

[13] Fujiwara Y, Heidelberger C. Fluorinated pyrimidines. 38. The incorporation of 5-trifluoromethyl-2’-deoxyuridine into the deoxyribonucleic acid of vaccinia virus. Mol Pharm. 1970;6:281–91. 5443533

[14] Fukushima M, Suzuki N, Emura T, Yano S, Kazuno H, Tada Y, et al. Structure and activity of specific inhibitors of thymidine phosphorylase to potentiate the function of antitumor 2’-deoxyribonucleosides. Biochem Pharm. 2000;59:1227–36. 10736423 10.1016/s0006-2952(00)00253-7

[15] Xu J, Kim TW, Shen L, Sriuranpong V, Pan H, Xu R, et al. Results of a Randomized, Double-Blind, Placebo-Controlled, Phase III Trial of Trifluridine/Tipiracil (TAS-102) Monotherapy in Asian Patients With Previously Treated Metastatic Colorectal Cancer: The TERRA Study. J Clin Oncol. 2018;36:350–8. 29215955 10.1200/JCO.2017.74.3245

[16] Nukatsuka M, Nakagawa F, Saito H, Sakata M, Uchida J, Takechi T. Efficacy of combination chemotherapy using a novel oral chemotherapeutic agent, TAS-102, with irinotecan hydrochloride on human colorectal and gastric cancer xenografts. Anticancer Res. 2015;35:1437–45. 25750295

[17] Tsukihara H, Nakagawa F, Sakamoto K, Ishida K, Tanaka N, Okabe H, et al. Efficacy of combination chemotherapy using a novel oral chemotherapeutic agent, TAS-102, together with bevacizumab, cetuximab, or panitumumab on human colorectal cancer xenografts. Oncol Rep. 2015;33:2135–42. 25812794 10.3892/or.2015.3876 PMC4391594

[18] Temmink OH, Hoebe EK, Fukushima M, Peters GJ. Irinotecan-induced cytotoxicity to colon cancer cells in vitro is stimulated by pre-incubation with trifluorothymidine. Eur J Cancer. 2007;43:175–83. 17049227 10.1016/j.ejca.2006.08.022

[19] Kuboki Y, Nishina T, Shinozaki E, Yamazaki K, Shitara K, Okamoto W, et al. TAS-102 plus bevacizumab for patients with metastatic colorectal cancer refractory to standard therapies (C-TASK FORCE): an investigator-initiated, open-label, single-arm, multicentre, phase 1/2 study. Lancet Oncol. 2017;18:1172–81. 28760399 10.1016/S1470-2045(17)30425-4

[20] Pfeiffer P, Yilmaz M, Möller S, Zitnjak D, Krogh M, Petersen LN, et al. TAS-102 with or without bevacizumab in patients with chemorefractory metastatic colorectal cancer: an investigator-initiated, open-label, randomised, phase 2 trial. Lancet Oncol. 2020;21:412–20. 31999946 10.1016/S1470-2045(19)30827-7

[21] Yoshida Y, Yamada T, Kamiyama H, Kosugi C, Ishibashi K, Yoshida H, et al. Combination of TAS-102 and bevacizumab as third-line treatment for metastatic colorectal cancer: TAS-CC3 study. Int J Clin Oncol. 2021;26:111–7. 33083913 10.1007/s10147-020-01794-8

[22] Satake H, Kato T, Oba K, Kotaka M, Kagawa Y, Yasui H, et al. Phase Ib/II Study of Biweekly TAS-102 in Combination with Bevacizumab for Patients with Metastatic Colorectal Cancer Refractory to Standard Therapies (BiTS Study). Oncologist. 2020;25:e1855–e63. 32666647 10.1634/theoncologist.2020-0643 PMC8108052

[23] Prager GW, Taieb J, Fakih M, Ciardiello F, Van Cutsem E, Elez E, et al. Trifluridine-Tipiracil and Bevacizumab in Refractory Metastatic Colorectal Cancer. N. Engl J Med. 2023;388:1657–67. 37133585 10.1056/NEJMoa2214963

[24] Li J, Qin S, Xu R, Yau TC, Ma B, Pan H, et al. Regorafenib plus best supportive care versus placebo plus best supportive care in Asian patients with previously treated metastatic colorectal cancer (CONCUR): a randomised, double-blind, placebo-controlled, phase 3 trial. Lancet Oncol. 2015;16:619–29. 25981818 10.1016/S1470-2045(15)70156-7

[25] Dasari A, Lonardi S, Garcia-Carbonero R, Elez E, Yoshino T, Sobrero A, et al. Fruquintinib versus placebo in patients with refractory metastatic colorectal cancer (FRESCO-2): an international, multicentre, randomised, double-blind, phase 3 study. Lancet. 2023;402:41–53. 37331369 10.1016/S0140-6736(23)00772-9

[26] Matsuoka H, Yamada T, Ohta R, Yoshida Y, Watanabe T, Takahashi M, et al. Biweekly TAS-102 and bevacizumab as third-line chemotherapy for advanced or recurrent colorectal cancer: a phase II, multicenter, clinical trial (TAS-CC4 study). Int J Clin Oncol. 2022;27:1859–66. 36201089 10.1007/s10147-022-02243-4

[27] Kotani D, Kuboki Y, Horasawa S, Kaneko A, Nakamura Y, Kawazoe A, et al. Retrospective cohort study of trifluridine/tipiracil (TAS-102) plus bevacizumab versus trifluridine/tipiracil monotherapy for metastatic colorectal cancer. BMC Cancer. 2019;19:1253. 31881856 10.1186/s12885-019-6475-6 PMC6935149

[28] Fujii H, Matsuhashi N, Kitahora M, Takahashi T, Hirose C, Iihara H, et al. Bevacizumab in Combination with TAS-102 Improves Clinical Outcomes in Patients with Refractory Metastatic Colorectal Cancer: A Retrospective Study. Oncologist. 2020;25:e469–e76. 32162797 10.1634/theoncologist.2019-0541 PMC7066722

[29] Matsuhashi N, Takahashi T, Fujii H, Suetsugu T, Fukada M, Iwata Y, et al. Combination chemotherapy with TAS-102 plus bevacizumab in salvage-line treatment of metastatic colorectal cancer: A single-center, retrospective study examining the prognostic value of the modified Glasgow Prognostic Score in salvage-line therapy of metastatic colorectal cancer. Mol Clin Oncol. 2019;11:390–6. 31475067 10.3892/mco.2019.1899 PMC6713946

[30] Yoshino T, Mizunuma N, Yamazaki K, Nishina T, Komatsu Y, Baba H, et al. TAS-102 monotherapy for pretreated metastatic colorectal cancer: a double-blind, randomised, placebo-controlled phase 2 trial. Lancet Oncol. 2012;13:993–1001. 22951287 10.1016/S1470-2045(12)70345-5

[31] Muro K, Boku N, Shimada Y, Tsuji A, Sameshima S, Baba H, et al. Irinotecan plus S-1 (IRIS) versus fluorouracil and folinic acid plus irinotecan (FOLFIRI) as second-line chemotherapy for metastatic colorectal cancer: a randomised phase 2/3 non-inferiority study (FIRIS study). Lancet Oncol. 2010;11:853–60. 20708966 10.1016/S1470-2045(10)70181-9

[32] Tabernero J, Yoshino T, Cohn AL, Obermannova R, Bodoky G, Garcia-Carbonero R, et al. Ramucirumab versus placebo in combination with second-line FOLFIRI in patients with metastatic colorectal carcinoma that progressed during or after first-line therapy with bevacizumab, oxaliplatin, and a fluoropyrimidine (RAISE): a randomised, double-blind, multicentre, phase 3 study. Lancet Oncol. 2015;16:499–508. 25877855 10.1016/S1470-2045(15)70127-0

[33] Van Cutsem E, Tabernero J, Lakomy R, Prenen H, Prausová J, Macarulla T, et al. Addition of aflibercept to fluorouracil, leucovorin, and irinotecan improves survival in a phase III randomized trial in patients with metastatic colorectal cancer previously treated with an oxaliplatin-based regimen. J Clin Oncol. 2012;30:3499–506. 22949147 10.1200/JCO.2012.42.8201

[34] Doi T, Yoshino T, Fuse N, Boku N, Yamazaki K, Koizumi W, et al. Phase I study of TAS-102 and irinotecan combination therapy in Japanese patients with advanced colorectal cancer. Invest N. Drugs. 2015;33:1068–77. 10.1007/s10637-015-0271-1 PMC4768213 26163340

[35] Varghese AM, Cardin DB, Hersch J, Benson AB, Hochster HS, Makris L, et al. Phase I Study of Trifluridine/Tipiracil Plus Irinotecan and Bevacizumab in Advanced Gastrointestinal Tumors. Clin Cancer Res. 2020;26:1555–62. 31924737 10.1158/1078-0432.CCR-19-2743

[36] Taniguchi H, Yamazaki K, Masuishi T, Kawakami T, Onozawa Y, Honda K, et al. Bevacizumab, Irinotecan, and Biweekly Trifluridine/Tipiracil for Metastatic Colorectal Cancer: MODURATE, a Phase Ib Study. Oncologist. 2023;28:e1108–e13. 37284901 10.1093/oncolo/oyad143 PMC10628564

[37] Gandaglia G, Karakiewicz PI, Briganti A, Passoni NM, Schiffmann J, Trudeau V, et al. Impact of the Site of Metastases on Survival in Patients with Metastatic Prostate Cancer. Eur Urol. 2015;68:325–34. 25108577 10.1016/j.eururo.2014.07.020

[38] Wang F, Jin Y, Wang M, Luo HY, Fang WJ, Wang YN, et al. Combined anti-PD-1, HDAC inhibitor and anti-VEGF for MSS/pMMR colorectal cancer: a randomized phase 2 trial. Nat Med. 2024;30:1035–43. 38438735 10.1038/s41591-024-02813-1

